# Vat Photopolymerization of Ceramic Parts: Effects of Carbon Fiber Additives on Microstructure and Mechanical Performance

**DOI:** 10.3390/ma17133127

**Published:** 2024-06-26

**Authors:** Lu Wang, Hailong Wu, Anfu Guo, Dekun Kong, Zhengyu Zhao, Chang Liu, Lvfa Yin, Guojun Xia, Xiaofei Su, Zhong Chen, Diangang Wang

**Affiliations:** 1 School of Mechanical and Automotive Engineering, Liaocheng University, Liaocheng 252000, China; 2021400315@stu.lcu.edu.cn (L.W.);; 2 Jiangsu Key Laboratory of Advanced Manufacturing Technology, Huaiyin Institute of Technology, Huaiyin 223003, China; 3 School of Materials Science and Engineering, Shandong University, Jinan 250061, China

**Keywords:** vat photopolymerization, ceramics, carbon fiber additives, microstructure, mechanical performance

## Abstract

Vat photopolymerization (VPP), as an additive manufacturing (AM) technology, can conveniently produce ceramic parts with high resolution and excellent surface quality. However, due to the inherent brittleness and low toughness of ceramic materials, manufacturing defect-free ceramic parts remains a challenge. Many researchers have attempted to use carbon fibers as additives to enhance the performance of ceramic parts, but these methods are mostly applied in processes like fused deposition modeling and hot pressing. To date, no one has applied them to VPP-based AM technology. This is mainly because the black carbon fibers reduce laser penetration, making it difficult to cure the ceramic slurry and thus challenging to produce qualified ceramic parts. To address this issue, our study has strictly controlled the amount of carbon fibers by incorporating trace amounts of carbon fiber powder into the original ceramic slurry with the aim to investigate the impact of these additions on the performance of ceramic parts. In this study, ceramic slurries with three different carbon fiber contents (0 wt.%, 0.1 wt.%, 0.2 wt.%, and 0.3 wt.%) were used for additive manufacturing. A detailed comparative analysis of the microstructure, physical properties, and mechanical performance of the parts was conducted. The experimental results indicate that the 3D-printed alumina parts with added carbon fibers show varying degrees of improvement in multiple performance parameters. Notably, the samples prepared with 0.2 wt.% carbon fiber content exhibited the most significant performance enhancements.

## 1. Introduction

Alumina (Al_2_O_3_), as a typical ceramic material, not only possesses high hardness, strength, and stability [1,2,3] but also exhibits excellent thermal, optical, and electromagnetic properties [4,5,6]. It is now widely used in industrial, medical, chemical, and aerospace fields [7,8,9]. Traditional Al_2_O_3_ ceramic preparation technologies primarily include molding, tape casting, injection molding [10], and gel casting [11]. Although the aforementioned technologies allow for rapid forming and meet the requirements for mass production, these traditional manufacturing techniques necessitate the pre-production of corresponding molds, which are expensive and complex. They are not suitable for non-standard parts or parts that are not intended for mass production, and they pose difficulties in fabricating components with complex internal structures, such as lattice structures and hollow structures. However, the emergence of a new additive manufacturing technology has overcome this inherent deficiency in the production process of traditional ceramic products [12]. Among all additive manufacturing (AM) technologies, Vat photopolymerization (VPP) can produce parts with complex shapes and structures with high precision and efficiency without the need for molds [13].

Although there are various ceramic preparation processes, the mechanical properties such as flexural strength, compressive strength, and toughness of ceramic parts still need improvement. Previously, extensive research has been conducted on this topic, including the addition of additives and the improvement of manufacturing processes, to enhance the durability and reliability of ceramic materials and expand their application scope. Through a literature review, it has been found that incorporating small amounts of carbon fibers into ceramic materials can significantly enhance their mechanical properties [14]. Due to the low quantity of carbon fibers used, the cost is also minimal. Currently, Baljinder Kandola et al., have investigated the thermal protection of ceramic particles in carbon fiber-reinforced composite materials [15]. Wolfgang Freudenberg et al., have studied the microstructure and mechanical properties of carbon fiber-reinforced ceramic matrix composites in AM technology based on filament melting [16]. Yuanhui Liu et al., have examined the influence of carbon fiber content on the microstructure, conductivity, and dielectric behavior of composite materials with Al_2_O_3_ ceramics in the hot-press sintering preparation process [17]. Virtudes Rubio et al., have researched the thermophysical properties of carbon fiber-reinforced ultra-high temperature ceramic matrix composites [18]. Haihua Wang et al., have explored the enhancing effect of short carbon fiber content and length on the mechanical properties of Al_2_O_3_ ceramic parts in ceramic printing-based AM technology [19]. The above-mentioned literature indicates that these approaches can improve the performance of ceramic products, but there are also many shortcomings. For example, although AM technology based on filament melting has lower costs, the parts produced often exhibit surface stacking patterns, leading to reduced strength. Ceramic parts produced by hot-press sintering have high precision and good mechanical properties, but they are difficult to fabricate into complex geometric shapes. AM technology based on extrusion has a faster forming speed but lower resolution, and the nozzle is prone to clogging. In contrast, VPP-based AM technology, which relies on the characteristics of photosensitive resin, has the advantage of layer-by-layer curing of ceramic slurry via laser irradiation. This technology allows for the production of complex geometric-shaped parts while ensuring high resolution and improved dimensional accuracy. The advantages and disadvantages of this method compared to other processes are shown in Table 1. Through a review of the literature, no studies have been found that utilize carbon fibers as additives to enhance the mechanical properties of ceramic parts using VPP-based AM technology. This is likely due to the black color of carbon fibers, which darkens the ceramic slurry, making it difficult for the slurry to be cured with a laser. Therefore, this study investigates the impact of carbon fiber additives on the performance of ceramic parts within the framework of VPP technology [20]. Given that higher carbon fiber content in the ceramic slurry results in greater difficulty in laser curing, it is necessary to minimize the amount of carbon fiber used. After multiple experiments, it was determined that the carbon fiber content should not exceed 0.3%, as higher concentrations lead to poor curing quality, making it challenging to produce ceramic samples. After batching, 3D printing, debinding, and sintering, ceramic parts with four different carbon fiber contents (0.0 wt.%, 0.1 wt.%, 0.2 wt.%, and 0.3 wt.%) were successfully fabricated. By analyzing the changes in their microstructure, physical properties, and mechanical properties, the impact of carbon fiber content on the performance of ceramic parts was thoroughly investigated. The analysis revealed that trace amounts of carbon fiber can significantly improve the microstructure and physical properties of the parts, leading to notable enhancements in their mechanical performance. Among them, ceramic parts with 0.2 wt.% carbon fiber showed the most significant performance improvement.

This study addresses a previously unexplored area by successfully incorporating carbon fiber powder as an additive in VPP-based AM technology, overcoming the significant challenge of laser penetration. This innovative approach results in the fabrication of Al_2_O_3_ parts with significantly enhanced strength, setting it apart from existing methods predominantly used in other manufacturing processes. By innovatively improving the production process of ceramic products, this study provides a novel approach to enhancing the various properties of ceramic materials. The improved quality of ceramic products can effectively expand their application scope, offering greater impetus to industries such as manufacturing, aerospace, medical devices, and electronics. Additionally, this research can provide new theoretical foundations and experimental methods for the development of AM technology.

## 2. Experiments and Measurement Procedures

### 2.1. Preparation of Raw Materials

**Figure 1:**
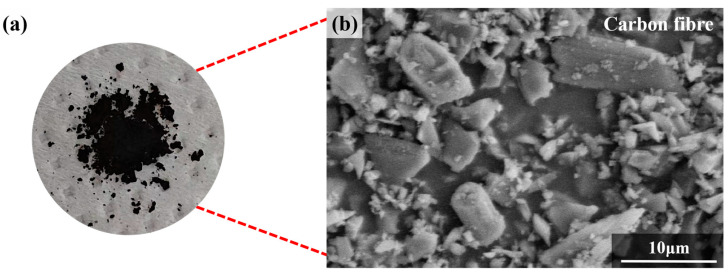
Carbon fiber powder: (**a**) Macroscopic image and (**b**) SEM micrograph.

The primary raw materials used in this study are α-Al_2_O_3_ powders (ZY-Al_2_O_3_-4, ZY-Al_2_O_3_-6, Hebei Chuancheng Metal Materials Co., Ltd., Xingtai, China), available in particle diameters of 100 nm and 500 nm. These powders are characterized by their low cost, high purity, high hardness, and excellent wear resistance. Additionally, their superior electrical insulation properties and uniform particle size distribution ensure material uniformity and high-quality finished products. The compositions are shown in Table 2. Other materials include magnesium oxide (MgO, Hebei Badu Metal Materials Co., Ltd., Xingtai, China), 1,6-hexanediol diacrylate (HDDA, Chengdu Sicheng Optoelectronic Materials Co., Ltd., Chengdu, China), trimethylolpropane triacrylate (TMPTA, Chengdu Sicheng Optoelectronic Materials Co., Ltd., Chengdu, China), epoxy resin (E51, Changzhou Runxiang Chemical Co., Ltd., Changzhou, China), and photoinitiator (1173, Chengdu Sicheng Optoelectronic Materials Co., Ltd., Chengdu, China). In ceramic additive manufacturing, HDDA, with the chemical formula C_16_H_32_O_4_, helps to make the slurry more uniform. TMPTA, with the chemical formula C_18_H_26_O_6_, increases the flowability of the slurry. E51, with the chemical formula (C_11_H_12_O_3_)_n_, has excellent adhesion and bonding capabilities, ensuring strong interlayer adhesion during the printing process. Meanwhile, 1173, with the chemical formula C_6_H_5_COC(CH_3_)_2_OH, enables the ceramic material to rapidly cure under specific wavelengths of light, allowing for layer-by-layer construction. Since the particle diameter of carbon fiber powder must be sufficiently fine to ensure effective curing [21], the additive used in this study is carbon fiber powder with a particle size of 800 mesh (Dongguan Wanlixiang Carbon Fiber Co., Ltd., Dongguan, China). When observed under an electron microscope, the particle structure of the carbon fiber powder appears columnar, as shown in Figure 1.

### 2.2. Preparation of Ceramic Slurry

**Figure 2:**
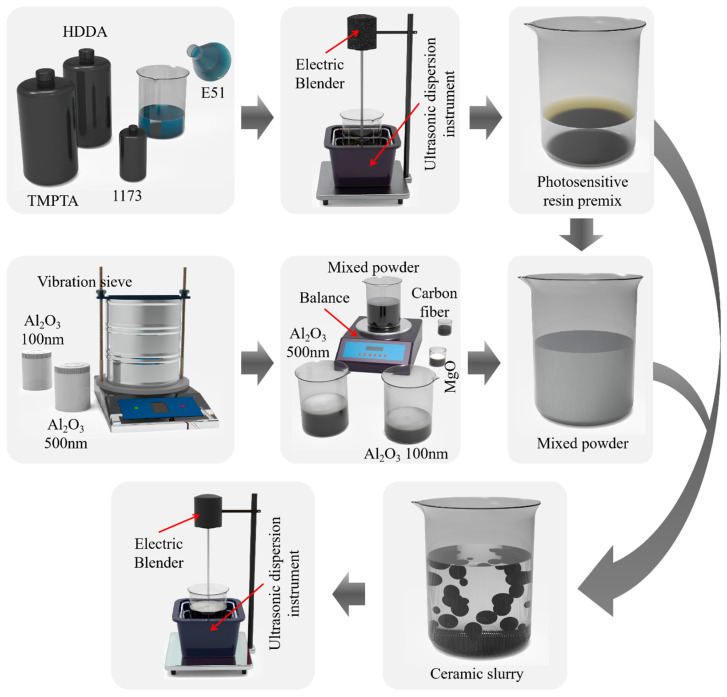
Preparation process.

The preparation of Al_2_O_3_ ceramic slurry involves three steps [22], as illustrated in Figure 2. The first step is the preparation of the photosensitive resin. HDDA, TMPTA, and E51 resin are initially mixed in a mass ratio of 1:1:1, followed by the addition of 1 wt.% photoinitiator (1173). The preliminary mixture is then stirred using an ultrasonic cleaner (JP-010 T, Shenzhen Jie Meng Cleaning Equipment Co., Ltd., Shenzhen, China) and an electric mixer (JJ-160 W, Jintan District Xicheng Xinrui Instrument Factory, Changzhou, China) for 20 min under ultrasonic conditions. The second step involves mixing the powder with the photosensitive resin. Al_2_O_3_ particles with diameters of 500 nm and 100 nm are initially mixed in a mass ratio of 4:1, followed by the addition of 1 wt.% magnesium oxide. This mixture is then combined with the photosensitive resin prepared in the first step at a ratio of 7:3 and stirred under ultrasonic conditions for 2 h to obtain the final Al_2_O_3_ ceramic slurry. The third step is the addition of the carbon fiber additive. Different mass fractions of carbon fiber (0 wt.%, 0.1 wt.%, 0.2 wt.%, and 0.3 wt.%) are added to the prepared Al_2_O_3_ ceramic slurry and stirred under ultrasonic conditions for 1 h to obtain three slurries with varying carbon fiber content. The ceramic slurry components are evenly distributed due to prolonged stirring with an electric mixer under ultrasonic conditions. Additionally, because the carbon fiber powder is black, mixing it with the ceramic slurry results in a darker color. This darker color absorbs light, which adversely affects the photopolymerization process of the ceramic [23]. Therefore, only a small amount of carbon fiber can be added. During practical experiments, it was found that when the carbon fiber content was 0.4 wt.%, the samples could not be formed due to poor curing performance. Therefore, only a small amount of carbon fiber can be added. Consequently, the experiment results in four types of ceramic slurry with carbon fiber contents of 0 wt.%, 0.1 wt.%, 0.2 wt.%, and 0.3 wt.%.

### 2.3. The Preparation of the Samples

**Figure 3:**
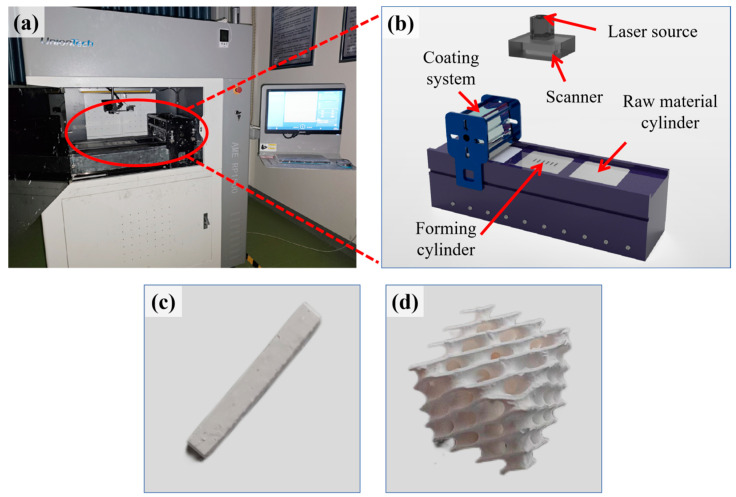
Fabrication of ceramic parts. (**a**) Stereolithography 3D printer, (**b**) working principle, (**c**) solid rectangular part, and (**d**) honeycomb-structured cubic part.

To fabricate ceramic parts using AM, the process begins with creating a 3D model using Solidworks. The model is then sliced using Voxeldance Additive 4.1.10 slicing software, and the resulting slice files are uploaded to the computer integrated into the stereolithography 3D printer (AME RP1500, UnionTech, Shanghai, China), as shown in Figure 3a. For this experiment, two distinct 3D models were designed: one is a solid rectangular prism with dimensions of 30 mm × 3 mm × 4 mm, as shown in Figure 3c; the other is a cube with a side length of 12 mm, featuring an internal honeycomb structure resembling a lattice, as shown in Figure 3d. In ceramic additive manufacturing, the selection of geometric shapes and dimensions for these two samples is based on previous research. The cuboid is the standard specimen for three-point bending tests. The choice of a honeycomb-patterned cube not only demonstrates the capability of additive manufacturing to create parts with complex internal structures but also facilitates mechanical and structural analysis. These shapes are widely used in material testing due to their simplicity and ease of fabrication and measurement. Before the actual printing, it is essential to carefully check and calibrate the equipment to ensure everything is functioning correctly. To accurately estimate the required printing time, a simulation print of the slice file is conducted. Once confirmed, the actual printing process can commence. The printer’s parameters for ceramic additive manufacturing are listed in Table 3. During printing, the laser source emits ultraviolet light to cure the ceramic slurry layer by layer, gradually building the preliminary ceramic green body, as shown in Figure 3b. After printing, the ceramic green body must be manually cleaned to remove any supports and excess slurry adhered to the surface of the part. To eliminate organic components and enhance the density and stability of the parts, the green ceramic bodies undergo debinding and sintering at high temperatures. To ensure the accuracy of the subsequent experimental data, 20 parts were produced for each of the two models at each concentration.

### 2.4. Debinding and Sintering

**Figure 4:**
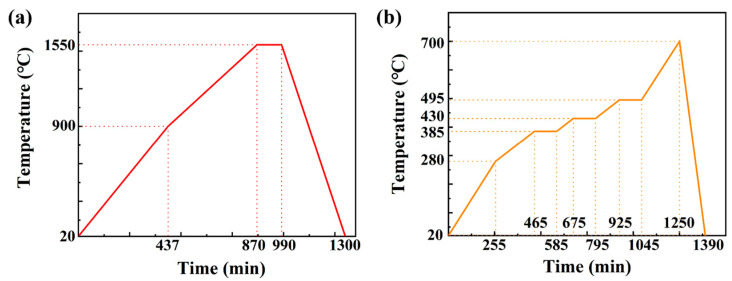
Temperature curves. (**a**) Debinding temperature curve and (**b**) sintering temperature curve.

Debinding is the process of removing organic components from the ceramic green body, while sintering is the process of bonding the ceramic particles together under high temperature to create a denser structure. In this experiment, both debinding and sintering were carried out using a muffle furnace (BR-17M, Bonahot Furnace Co., Ltd., Zhengzhou, China) under high-temperature conditions. During debinding, the ceramic green body is placed in the muffle furnace, where the temperature is gradually increased under controlled atmospheric conditions to volatilize the organic components. During sintering, the ceramic green body is placed in the muffle furnace at high temperatures, which further reduces the gaps between ceramic particles, resulting in a more robust ceramic structure [24]. Both debinding and sintering require setting appropriate parameters such as time and temperature on the control panel of the muffle furnace [25]. The specific temperature variation curve is shown in Figure 4.

### 2.5. Characterization

Ceramic green bodies fabricated using a stereolithography-based 3D printer were subjected to debinding and sintering to obtain ceramic parts with four different carbon fiber concentrations. From each of the two ceramic parts with different concentrations, 15 of the highest-quality, defect-free parts were selected as test samples. In subsequent experiments, each test was repeated at least five times, and the average values of the experimental data were calculated.

During the debinding and sintering processes, the organic materials in the ceramic samples were removed, and high-temperature treatment made the parts more dense, resulting in dimensional changes. To calculate the shrinkage rate, the dimensions of the samples were measured in the X, Y, and Z directions both before debinding and after sintering. The shrinkage rate was then calculated using the following formula [26].

(1) $$C=\frac{L_{1}-L_{2}}{L_{1}}\times 100\%$$

where *C* is the shrinkage rate (%), *L*_1_ is the dimension of the sample before debinding (mm), and *L*_2_ is the dimension of the sample in the same direction after sintering (mm).

According to Archimedes’ principle, the volume density, water absorption, and porosity of the samples were measured using an automatic electronic densitometer (Byes-300B, Bangyi Precision Instruments Co., Ltd., Shanghai, China) [27]. The relevant calculation formulas are as follows.

(2) $$D_{b}=\frac{G_{1}\times D_{w}}{G_{1}-G_{3}}$$

(3) $$W=\frac{G_{2}-G_{1}}{G_{1}}\times 100\%$$

(4) $$O=\frac{G_{2}-G_{1}}{G_{2}-G_{3}}\times 100\%$$

where *D_b_* is the volume density of the sample (g/cm^3^), *W* is the water absorption of the sample (%), *O* is the porosity of the sample (%), *D_w_* is the density of water (g/cm^3^), *G*_1_ is the mass of the dry sample (g), *G*_2_ is the mass of the wet sample (g), and *G*_3_ is the buoyant mass of the sample in water (g).

**Figure 5:**
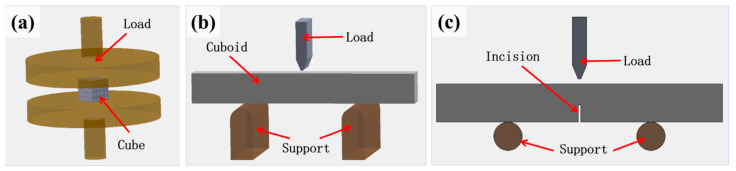
Schematic of mechanical tests. (**a**) Compression test, (**b**) three-point bending test, and (**c**) single-edge V-notch beam test.

To measure the mechanical properties of the samples, a computer-controlled universal testing machine (Yixuan Test Instrument Co., Ltd., Cangzhou, China) was used to perform three-point bending tests and uniaxial compression tests. To verify the changes in the mechanical properties of additively manufactured ceramic parts after the addition of carbon fibers, the compressive strength of cubic samples with complex internal structures was measured through uniaxial compression tests [28], as shown in Figure 5a. The flexural strength of rectangular samples was measured through three-point bending tests [29], as shown in Figure 5b. To minimize variability, three samples of each category were randomly selected for testing. After testing, the experimental data were organized, and stress, strain, Young’s modulus, and energy density (the area enclosed by the stress–strain curve and the *x*-axis) were calculated. The mechanical properties of each sample category were further analyzed based on these calculations.

For cubic samples with a honeycomb internal structure, the following formulas were used for the calculations:

(5) $$\sigma_{b}=\frac{F}{A}$$

(6) $$\varepsilon_{b}=\frac{\Delta H}{H_{0}}$$

where *σ_b_* is the uniaxial compressive stress (MPa), *F* is the applied force (N), *A* is the area of the maximum cross-section, *ε_b_* is the uniaxial compressive strain (%), Δ*H* is the change in sample thickness (mm), and *H*_0_ is the initial sample thickness (mm) (edge length of the cube).

For solid rectangular specimens, the following formula is used for calculation:

(7) $$\sigma_{a}=\frac{3FL}{2bh^{2}}$$

(8) $$\varepsilon_{a}=\frac{6D_{f}h}{L^{2}}$$

where *σ_a_* is the flexural stress (MPa), F is the applied load (N), L is the span length (mm), b is the width of the specimen (mm), h is the height of the specimen (mm), *ε_a_* is the flexural strain (%), and *D_f_* is the mid-span deflection (mm).

Based on the obtained stress and strain values, the corresponding stress–strain curves were plotted to calculate the Young’s modulus and energy density for each sample [30]. Young’s modulus measures the sample’s ability to resist deformation, while energy density reflects the material’s ability to absorb energy [31]. The relevant calculation formulas are as follows:

(9) $$E=\frac{\Delta \sigma }{\Delta \varepsilon }$$

(10) $$U=\int_{a}^{b}f\left(x\right)dx$$

where *E* represents Young’s modulus, Δ*σ* is the stress increment within a specified range, and Δ*ε* is the corresponding strain increment. The energy absorption density *U* is defined over the strain interval [a, b] used for energy absorption testing, with *f(x)* representing the fitted stress–strain curve.

To measure the toughness of the sintered samples, the single-edge V-notched beam (SEVNB) method was employed [32], as illustrated in Figure 5c. A 200 µm thick low-speed diamond blade was used to saw a 2 mm deep notch in the center of a rectangular sample measuring 3 mm in width and 4 mm in height. The sample was then placed in a universal testing machine. The machine, controlled using computer software, applied pressure to the sample while monitoring the fracture behavior during loading, recording load and displacement data. The fracture toughness was calculated using the following formula:

(11) $$K_{IC}=\frac{3PL}{2bh^{1.5}}\cdot {\left(\frac{a}{h}\right)}^{0.5}\cdot \frac{1.99-\frac{a}{h}\times \left(1-\frac{a}{h}\right)\left[2.15-3.93\cdot \frac{a}{h}+2.7{\left(\frac{a}{h}\right)}^{2}\right]}{\left(1+2\cdot \frac{a}{h}\right){\left(1-\frac{a}{h}\right)}^{1.5}}$$

where *P* is the applied load (N), and *h* is the notch depth (mm).

To observe the surface morphology of the samples, a dual-beam focused ion beam scanning electron microscope (FIB-SEM) (GX4, Thermo Fisher, Waltham, MA, USA) was utilized to examine the microstructure of the sample surfaces [33]. Additionally, the FIB-SEM’s integrated EDS system was employed to analyze the elemental distribution and composition of the materials. To determine the phase composition of the crystalline materials in the samples, an X-ray diffractometer (XRD, PANalytical B.V, Almelo, Netherlands) was used to analyze the material’s composition, with a testing range of 5° to 90° and a scanning speed of 2°/min. To observe the crystalline phases, grain sizes, and defect conditions of the samples, an inVia confocal laser Raman spectrometer (InVia, Renishaw, London, UK) was used to collect the Raman spectra of the sample powders, with a laser source wavelength of 532 nm, a power of 50 mW, and a slit width of 50 µm [34]. For characterizing the surface elements and their chemical states, X-ray photoelectron spectroscopy (XPS) (EscaLab Xi+, Thermo Fisher, Waltham, MA, USA) was performed to conduct qualitative analysis of the chemical elements in the sample powders [35].

## 3. Results and Discussion

### 3.1. Effect on Microstructure

**Figure 6:**
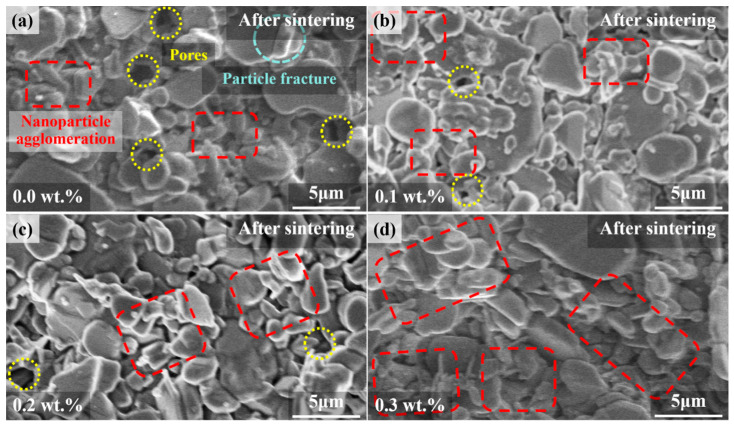
SEM micrographs of sintered samples. (**a**) Sample without carbon fiber, (**b**) sample with 0.1 wt.% carbon fiber, (**c**) sample with 0.2 wt.% carbon fiber, and (**d**) sample with 0.3 wt.% carbon fiber.

The microstructural images of the samples obtained through scanning electron microscopy (SEM) are presented in Figure 6a–d. From the SEM images of the sintered samples, it is evident that the samples without added carbon fiber exhibit higher porosity, fewer aggregated fine particles, and instances of intergranular fracture. Intergranular fracture indicates that cracks propagate along the grain boundaries, which reduces the material’s strength [36]. Conversely, the samples containing carbon fiber show fewer pores, more aggregated particles, and less particle fracture [37]. This phenomenon may be attributed to the transition of carbon fibers from a solid phase to a liquid phase during the sintering process. This suggests that Al_2_O_3_ parts with carbon fiber are denser, which helps improve their mechanical properties. Comparative analysis of the images of samples with different carbon fiber contents reveals that the samples with 0.2 wt.% and 0.3 wt.% carbon fiber exhibit the best quality. This is likely because, during the sintering process [38], the particles in carbon fiber-containing samples exhibit stronger adsorption forces. These forces facilitate the aggregation of fine particles and prevent intergranular fracture of larger grains.

**Figure 7:**
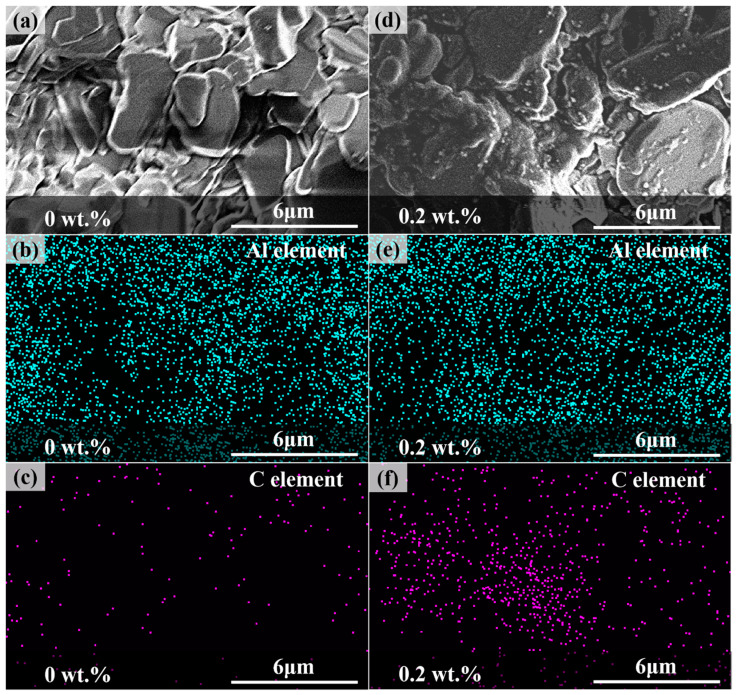
EDS images. (**a**) Testing area of the sample without carbon fibers, (**b**) distribution of Al element, (**c**) distribution of C element, (**d**) testing area of the sample with 0.2 wt.% carbon fibers, (**e**) distribution of Al element, and (**f**) distribution of C element.

To demonstrate the presence and effect of carbon fibers, the EDS system was used to measure the distribution of Al and C elements in both the sample without additives and the sample with 0.2 wt.% carbon fiber, as shown in Figure 7. Figure 7a,b show that the surface of the sample without additives is clean and smooth, while the surface of the sample containing carbon fiber is coated with a layer of material. Figure 7c,d indicate that the Al element distribution is more uniform in the sample with 0.2 wt.% carbon fiber. Figure 7e,f reveal a significant increase in the C element in the sample containing carbon fiber. This indicates that carbon fibers can improve the flowability of the ceramic slurry, reducing the occurrence of voids and defects during the photocuring process. Consequently, this leads to a more uniform distribution of Al elements during the curing process [39]. A uniform distribution of Al elements can reduce stress concentration, enhancing the mechanical properties of Al_2_O_3_ parts. Additionally, during the sintering process, carbon fibers transition from a solid to a liquid phase, so they are no longer in a fibrous structure. After sintering, the carbon fibers in the liquid phase fully adhere to the pore surfaces within the ceramic material, thereby increasing the material’s strength [40].

**Figure 8:**
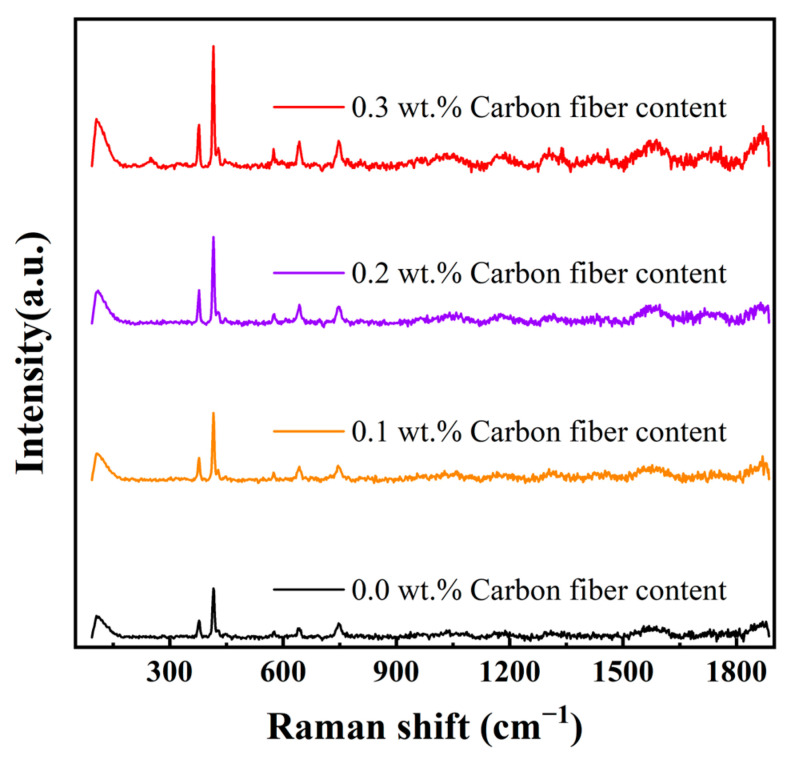
The measured Raman spectra of the four samples.

The crystal structure of the ceramic parts was analyzed using a Raman spectrometer. The Raman spectra of Al_2_O_3_ samples with varying carbon fiber content are shown in Figure 8. The peaks associated with α-Al_2_O_3_ are located around 377 cm^−1^, 415 cm^−1^, 428 cm^−1^, 447 cm^−1^, 574 cm^−1^, 641 cm^−1^, and 747 cm^−1^. As the carbon fiber content increases, the characteristic peaks in the Raman spectra become stronger and sharper. This indicates that the addition of carbon fiber powder facilitates the growth of regular crystal grains, leading to an increase in crystallinity. Consequently, the molecular chains are more orderly arranged, which enhances structural stability and reliability [41].

**Figure 9:**
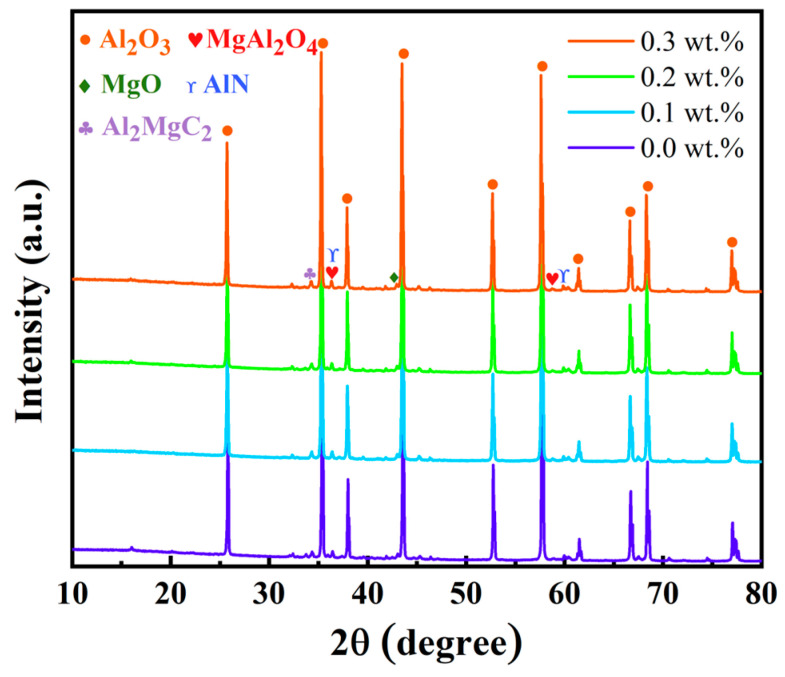
The comparison of XRD peaks of the four samples.

The XRD patterns obtained from the X-ray diffractometer are shown in Figure 9. The patterns reveal that the primary component of the samples is Al_2_O_3_, with minor quantities of Al_2_MgC_2_ and AlN present. There are no significant differences in the corresponding peaks of these three substances across the four different samples. The magnesium aluminate (MgAl_2_O_4_) and AlN phases are formed through chemical reactions during the sintering process [42]. Notably, the peak corresponding to MgAl_2_O_4_ near 58° is more pronounced in the samples containing carbon fiber. The formation of MgAl_2_O_4_ is facilitated by the carbon fibers creating a conductive network within the samples at high temperatures, allowing reactants to come into closer contact and react more efficiently, thereby increasing the yield of MgAl_2_O_4_ [43]. MgAl_2_O_4_ acts as a sintering aid, enhancing the quality of the sintering process and contributing to the improvement of the material’s hardness and thermal stability [44].

**Figure 10:**
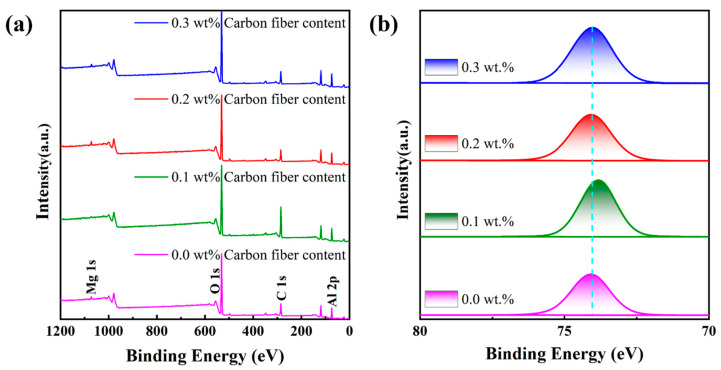
XPS spectra. (**a**) Full spectrum and (**b**) high-resolution spectrum of Al element.

The chemical elemental composition of the sintered samples was characterized using XPS, as shown in Figure 10. The peak at 284.8 eV corresponds to the presence of carbon [45], which can be attributed to two main factors: first, prolonged exposure of the sample to air, which contains carbon; and second, the carbon fiber additive introduced during the process. Analysis reveals that, except for a slight rightward shift in the binding energy of the sample with 0.1% carbon fiber, the binding energies of the other samples show no significant change. This indicates that the addition of carbon fiber does not increase the binding energy of Al elements. For the C element, the peak at 284.8 eV is notably higher in samples containing carbon fiber, and the peak intensity increases with the carbon fiber content, consistent with the results of the EDS and XRD analyses. Additionally, the peak at 74.6 eV corresponds to Al, with its fine spectrum shown in Figure 10b.

### 3.2. Effect on Physical Properties

**Figure 11:**
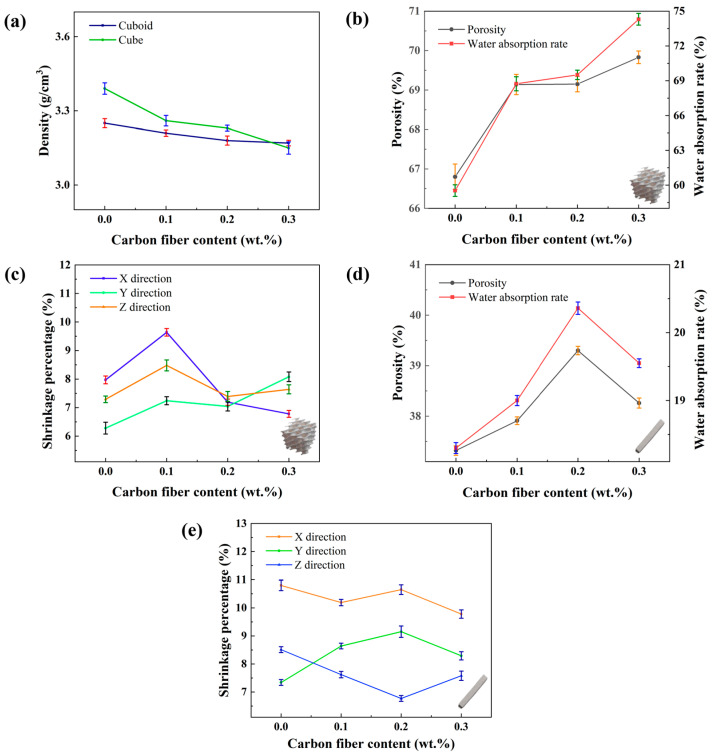
Influence of carbon fiber content on physical properties. (**a**) Density variation curves of rectangular and cubic samples, (**b**) shrinkage rate variation curve of rectangular samples, (**c**) shrinkage rate variation curve of cubic samples, (**d**) porosity and water absorption variation curves of rectangular samples, and (**e**) porosity and water absorption variation curves of cubic samples.

By measuring the mass and calculating the density, it was observed that both the mass and density of the samples decreased with the increase in carbon fiber content. The variation curves of mass and density with carbon fiber content are shown in Figure 11a. There are two main reasons for the density decrease: first, the lower density of carbon fibers reduces the overall density of the slurry, thereby decreasing the sample density; second, the addition of carbon fibers darkens the slurry, affecting the photopolymerization process and consequently altering the sample density. The reduction in density results in a lower mass for the same volume model, which is advantageous for manufacturing lightweight products.

Regarding dimensional changes, the actual size of the freshly prepared ceramic green body tends to be slightly larger than the specified size, with an error margin not exceeding 5%. Compared to the samples without additives, those with added carbon fibers are slightly smaller in size both before debinding and after sintering. This is likely due to the addition of carbon fibers altering the composition and density of the ceramic slurry, thereby affecting the final dimensions of the parts. A comparative analysis of the sample sizes before debinding and after sintering indicates that the dimensions in all three directions decrease with increasing carbon fiber content. This reduction is attributed to the increased viscosity of the ceramic slurry with added carbon fibers, leading to a certain degree of shrinkage during the curing process. Although the dimensions of the samples with carbon fibers are slightly reduced, the shrinkage rate, calculated from the dimensions before debinding and after sintering shows no significant variation due to the carbon fiber addition. The changes in shrinkage rate with different carbon fiber contents are shown in Figure 11b,c.

The water absorption and porosity of the samples were calculated using respective formulas, with the results shown in Figure 11d,e. The calculations indicate that both water absorption and porosity increase in the samples containing additives. Specifically, the water absorption and porosity of the cubic samples increase with the carbon fiber content. For the rectangular samples, these properties increase with carbon fiber content up to 0.2 wt.% but decrease slightly at 0.3 wt.%, although still remaining higher than those of the samples without additives. This is likely due to the oxidation reaction of the carbon fibers on the surface during sintering, which creates small pores [46]. The increase in water absorption and porosity enhances the material’s breathability and sound absorption. To investigate whether the increased water absorption and porosity adversely affect the material’s strength, a series of mechanical tests were conducted for detailed analysis.

### 3.3. Effect on Mechanical Properties

To investigate the impact of carbon fiber additives on the mechanical properties of ceramic samples, compression tests and three-point bending tests were conducted to assess their mechanical performance. Additionally, the toughness of the samples was evaluated using the single-edge V-notched beam (SEVNB) method. The experimental results are as follows.

**Figure 12:**
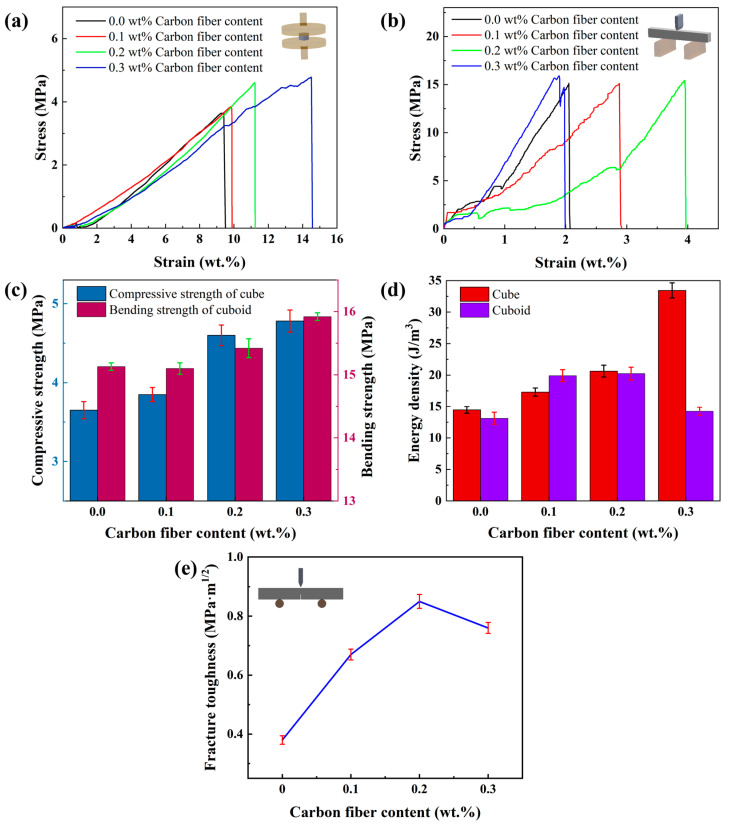
Influence of carbon fiber content on mechanical properties. (**a**) Stress–strain curve of compression test, (**b**) stress–strain curve of three-point bending test, (**c**) bending strength of rectangular prism and compression strength of cube, (**d**) energy absorption density, and (**e**) toughness.

Through compression tests on cube samples with internal honeycomb structures, it was found that the maximum compressive forces borne by samples with carbon fiber contents of 0 wt.%, 0.1 wt.%, 0.2 wt.%, and 0.3 wt.% were 525.79 N, 553.95 N, 662.79 N, and 687.72 N, respectively, increasing with the carbon fiber content. Stress and strain of the samples were calculated using Equations (5) and (6), and stress–strain curves were plotted, as shown in Figure 12a. The flexural strength obtained from the stress–strain curves is illustrated in Figure 12c. It can be observed from the graphs that samples containing carbon fibers exhibited slightly reduced curve slopes, leading to a decrease in Young’s modulus. While a lower Young’s modulus may not significantly enhance the mechanical properties of the material, it can impart characteristics such as flexibility and light weight [47], thereby aiding in stress reduction, consistent with the density test results in the physical properties section. However, in Figure 12a, the ceramic sample with 0.3 wt.% carbon fiber content shows negligible changes in stress but notable changes in strain. This phenomenon is mainly due to the darkening of the ceramic slurry with increasing carbon fiber content, which affects the photocuring process and leads to insufficient interlayer adhesion, thereby reducing the compressive strength of the cubic samples. Additionally, a lower Young’s modulus provides the material with some flexibility but does not significantly improve mechanical properties. Since the area enclosed by the curves and the *x*-axis increases sequentially, the energy density increases significantly with the carbon fiber content, as shown in Figure 12d. Consequently, ceramic components containing carbon fibers exhibited significantly improved energy absorption rates, enhancing their impact resistance and reducing the likelihood of brittle fracture [48,49].

Through three-point bending tests on solid rectangular prism samples, it was found that samples with carbon fiber contents of 0 wt.%, 0.1 wt.%, 0.2 wt.%, and 0.3 wt.% could withstand maximum bending forces of 32.27 N, 32.22 N, 32.89 N, and 33.97 N, respectively. Using Equations (7) and (8), the bending stress and strain of the samples were calculated, and stress–strain curves were plotted, as shown in Figure 12b. From the graph, it can be observed that the bending strength of the sample did not change significantly after the addition of carbon fibers. According to Equation (9), the Young’s modulus calculation results were consistent with those of the honeycomb-shaped cube mentioned earlier. According to Equation (10), the energy density increased with the increase in carbon fiber content, as depicted in Figure 12d. However, when the carbon fiber content reached 0.3 wt.%, the Young’s modulus increased while the energy density decreased. Additionally, the lower strain values for the 0.3 wt.% carbon fiber content samples in Figure 12b can be attributed to the photocuring process and the nature of the carbon fibers. As the carbon fiber content increases to 0.3 wt.%, the ceramic slurry becomes darker, resulting in greater absorption of laser energy during photocuring. Furthermore, an important factor is that Figure 12b represents solid rectangular samples, which require more slurry per layer to cure compared to non-solid cube samples. The increased absorption causes the surface layer to cure faster and the deeper layers to cure incompletely. This incomplete curing introduces residual stresses and slight, imperceptible cracks in the samples post-debinding and sintering [50], adversely affecting the mechanical properties and reducing the overall strain observed in the 0.3 wt.% carbon fiber samples compared to those with 0.1 wt.% and 0.2 wt.% carbon fibers.

Figure 12e illustrates the fracture toughness of Al_2_O_3_ components obtained through the single-edge V-notch beam method, experimentally determined and calculated. It is evident from the graph that components containing carbon fibers exhibit higher toughness, with Al_2_O_3_ components containing 0.2 wt.% carbon fibers demonstrating the highest toughness.

In summary, the addition of carbon fiber additives has the most significant effect on improving the mechanical properties of the cubic samples, with both compression strength and toughness increasing with carbon fiber content. Conversely, the improvement in mechanical properties of the rectangular prism samples with carbon fiber additives is less pronounced, especially when the carbon fiber content reaches 0.3 wt.%, as the mechanical properties of the samples weaken. This is because the cubic samples have a honeycomb internal structure, with less ceramic slurry used during layer-by-layer curing, resulting in better curing effects and less susceptibility to factors such as laser absorption by carbon fibers. In contrast, the rectangular prism samples have a solid internal structure, longer curing times, and are more susceptible to factors such as laser absorption by carbon fibers, resulting in weaker mechanical properties due to poor curing effects. Therefore, for VPP-based AM technology, ceramic slurries containing carbon fibers are more suitable for producing thin-walled components.

## 4. Conclusions

In this study, we successfully fabricated four types of ceramic parts with varying carbon fiber content (0 wt.%, 0.1 wt.%, 0.2 wt.%, and 0.3 wt.%) using VPP-based AM technology. The research demonstrates that the addition of carbon fibers can significantly improve the microstructure and mechanical properties of ceramic parts, particularly for those with 0.2 wt.% carbon fiber content. The key findings are summarized as follows.

In terms of microstructure, samples containing carbon fibers exhibited fewer surface defects and more densely packed grains. Regarding mechanical properties, the compressive strength increased by up to 31.0%, and toughness improved by up to 43.1%, while the flexural strength showed no significant change. The enhancement in mechanical properties helps prevent deformation and fracture of ceramic products. Additionally, in terms of physical properties, parts with carbon fibers became smaller in size after sintering, with reduced density, increased water absorption, and higher porosity. This indicates that carbon fiber-reinforced ceramic parts are lightweight, breathable, and absorptive.

In summary, carbon fiber-reinforced ceramic parts offer numerous advantages and can be widely used in aerospace, medical devices, and electronics. This study successfully enhanced the performance of ceramic parts by overcoming laser penetration issues, innovatively improving the production process of ceramic products. This approach provides a new method for enhancing the performance of ceramic products, distinguishing it from other common manufacturing processes. The improved quality of ceramic products can effectively expand their application range and provide new theoretical and experimental methods for the development of AM technology.

## Figures and Tables

**Table 1:** Comparison of advantages and disadvantages of different preparation processes.

Preparation Process	Microstructure	Mechanical Properties	Forming Accuracy	Manufacturing Complex Structures
Fused-Filament Fabrication AM Technology	Layered Lines	Slightly Enhanced	High Accuracy	Supported
Hot-Press Sintering	Few Defects	Significantly Enhanced	Moderate Accuracy	Not supported
Extrusion-Based AM Technology	Few Defects	Significantly Enhanced	Moderate Accuracy	Supported
VPP-Based AM Technology	Few Defects	Significantly Enhanced	High Accuracy	Supported

**Table 2:** The elemental composition and mass fraction of two types of alumina powders.

Grain Size	Element	Al_2_O_3_	Na_2_O	Fe_2_O_3_	TiO_2_	K_2_O	SiO_2_	CaO	MgO
100 nm	wt.%	≥99.9	0.02	0.01	0.01	0.01	<0.01	0.02	<0.01
500 nm	wt.%	≥99.9	0.015	0.026	0.01	0.009	0.02	-	-

**Table 3:** Printing parameters.

Wavelength	Layer Thickness	Laser Power	Fill Distance	Fill Pattern
355 nm	50 μm	120 mW	30 μm	X-Y

## Data Availability

Data are contained within the article.
